# Genomic evidence for dysregulated glutamine metabolism in the asthmatic airway epithelium

**DOI:** 10.1002/clt2.12178

**Published:** 2022-07-05

**Authors:** Daniela C. Bravo‐Solarte, Kimberly E. Stelzig, Lyda Cuervo‐Pardo, Sergejs Berdnikovs, Sergio E. Chiarella

**Affiliations:** ^1^ Division of Allergic Diseases Mayo Clinic Rochester Minnesota USA; ^2^ Department of Anesthesiology and Perioperative Medicine Mayo Clinic Rochester Minnesota USA; ^3^ Division of Rheumatology, Allergy and Clinical Immunology University of Florida Gainesville Florida USA; ^4^ Division of Allergy and Immunology Northwestern University Feinberg School of Medicine Chicago Illinois USA

**Keywords:** allergy, asthma, sports

To the Editor,

Airway epithelial cells (AECs) play a critical role in lung tissue homeostasis and disease. In asthma, AECs can promote the development of airway inflammation and remodeling through the secretion and regulation of a wide array of mediators, such as arachidonic acid metabolites, growth factors, cytokines, and chemokines.^1^ As discussed in a recent review, the dysregulation of metabolic pathways in AECs is highly relevant to asthma.^2^ Even though glutamine metabolism is key to multiple biochemical pathways, it has remained understudied. In the present study, we sought to identify genes related to glutamine metabolism that are differentially expressed in patients with severe or T helper 2 (Th2)‐high asthma when compared to healthy controls (HCs).

Our group performed a secondary analysis of two publicly available microarray datasets involving fresh bronchial brushing samples. We selected these two datasets as they were available in the National Center for Biotechnology Information's Gene Expression Omnibus (accession numbers GSE43696 and GSE67472), had well‐documented clinical description of asthmatics and airway sampling procedures, included samples from HCs, and were supported by peer‐reviewed PubMed‐indexed publications.^3^, ^4^ In addition, both datasets had sufficient samples from asthmatics and HCs, which enabled us to generate a list of differentially expressed genes after false discovery rate (FDR) significance adjustment.

The first dataset (GSE43696) included 38 individuals with severe asthma and 20 HCs. Subjects were determined to have severe asthma based on inhaled or oral corticosteroid use and/or airflow limitation.^4^ These samples were processed using the Whole Human Genome Microarray 4 × 44 K (Agilent). The second dataset (GSE67472) included 40 patients with Th2‐high asthma and 43 HCs. Subjects were determined to have Th2‐high asthma based on the expression levels of three interleukin (IL)‐13‐inducible genes in AECs: periostin, serine peptidase inhibitor B2 (SERPINB2), and chloride channel Ca^2+^‐activated 1 (CLCA1).^3^ In this case, the samples were processed using the Human Genome U133 Plus 2.0 Array (Affimetrix). For comparisons between the asthmatic and HC groups, we performed moderated Benjamini‐Hochberg *t*‐tests (with FDR significance adjustment) and identified all the differentially expressed genes (*p*‐value <0.05). Finally, to identify the genes involved in glutamine metabolism we performed a literature search in PubMed and the Molecular Signatures Database (MSigDB). We included genes involved in the anabolic, catabolic, and transport processes of glutamine.

We identified 11 genes that were differentially expressed in both cohorts and showed the same directionality in asthmatics versus HCs (Table 1). Within this group of genes involved in glutamine metabolism, the ones that were upregulated in the AECs of asthmatics from both datasets include pyrroline‐5‐carboxylate reductase 1 (PYCR1), glutathione peroxidase 7 (GPX7), glutamic‐oxaloacetic transaminase 1 (GOT1), glutamic‐oxaloacetic transaminase 2 (GOT2), phosphoserine aminotransferase 1 (PSAT1), solute carrier family 7 member 11 (SLC7A11), nitric oxide synthase 2 (NOS2), solute carrier family 39 member 8 (SLC39A8), and sirtuin 7 (SIRT7). The two genes that were downregulated in AECs of the asthmatics from both datasets were glutaminase (GLS) and O‐linked N‐acetylglucosamine (GlcNAc) transferase (OGT).

**TABLE 1:** Genes involved in glutamine metabolism that are differentially expressed in both cohorts of asthmatics

Gene symbol	Gene name	Asthmatics versus healthy controls	Adjusted *p*‐value [GSE43696]	Log_2_ (fold change) [GSE43696]	Adjusted *p*‐value [GSE67472]	Log_2_ (fold change) [GSE67472]
PYCR1	Pyrroline‐5‐carboxylate reductase 1	Increased	0.00325344	0.93739012	0.0000931	0.22801566
GLS	Glutaminase	Decreased	0.00372665	−0.3316336	0.119	−0.12946669
GPX7	Glutathione peroxidase 7	Increased	0.01883538	0.52443388	0.00223	0.22698268
OGT	O‐linked N‐acetylglucosamine (GIcNAc) transferase	Decreased	0.03984839	−0.27940922	0.0202	−0.13742996
SLC39A8	Solute carrier family 39 member 8	Increased	0.05686103	0.54797585	0.00000000487	0.36324071
NOS2	Nitric oxide synthase 2	Increased	0.05710097	1.40178768	0.00000000139	0.64266396
GOT2	Glutamic‐oxaloacetic transaminase 2	Increased	0.07886384	0.26489575	0.000729	0.23846269
SLC7A11	Solute carrier family 7 member 11	Increased	0.12690056	0.44360264	0.00723	0.17956673
GOT1	Glutamic‐oxaloacetic transaminase 1	Increased	0.20214456	0.13258827	0.0143	0.19680155
PSAT1	Phosphoserine aminotransferase 1	Increased	0.16001014	0.38265011	0.0119	0.21649468
SIRT7	Sirtuin 7	Increased	0.2861937	0.12896795	0.000178	0.25160105

Glutamine has pleiotropic effects on cell metabolism. Strikingly, our results shows that there are multiple genes involved in glutamine metabolism that are differentially expressed in asthmatic AECs (Figure 1). For instance, in both datasets, we identified lower levels of GLS expression in AECs of asthmatics versus HCs. This is consistent with a prior report that demonstrated, through immunohistochemistry, decreased levels of GLS in the lung biopsy of a patient with severe asthma.^5^ Importantly, GLS catalyzes the first obligatory step in glutaminolysis to generate glutamate and ammonia. The ammonia generated through this reaction can serve as a buffer for airway pH.^5^, ^6^ Increased airway acidity can promote the conversion of nitrite to nitric oxide (NO) and accelerate eosinophil necrosis, which can trigger airway inflammation in asthma. Therefore, decreased levels of GLS could significantly contribute to lung inflammation.

**FIGURE 1:**
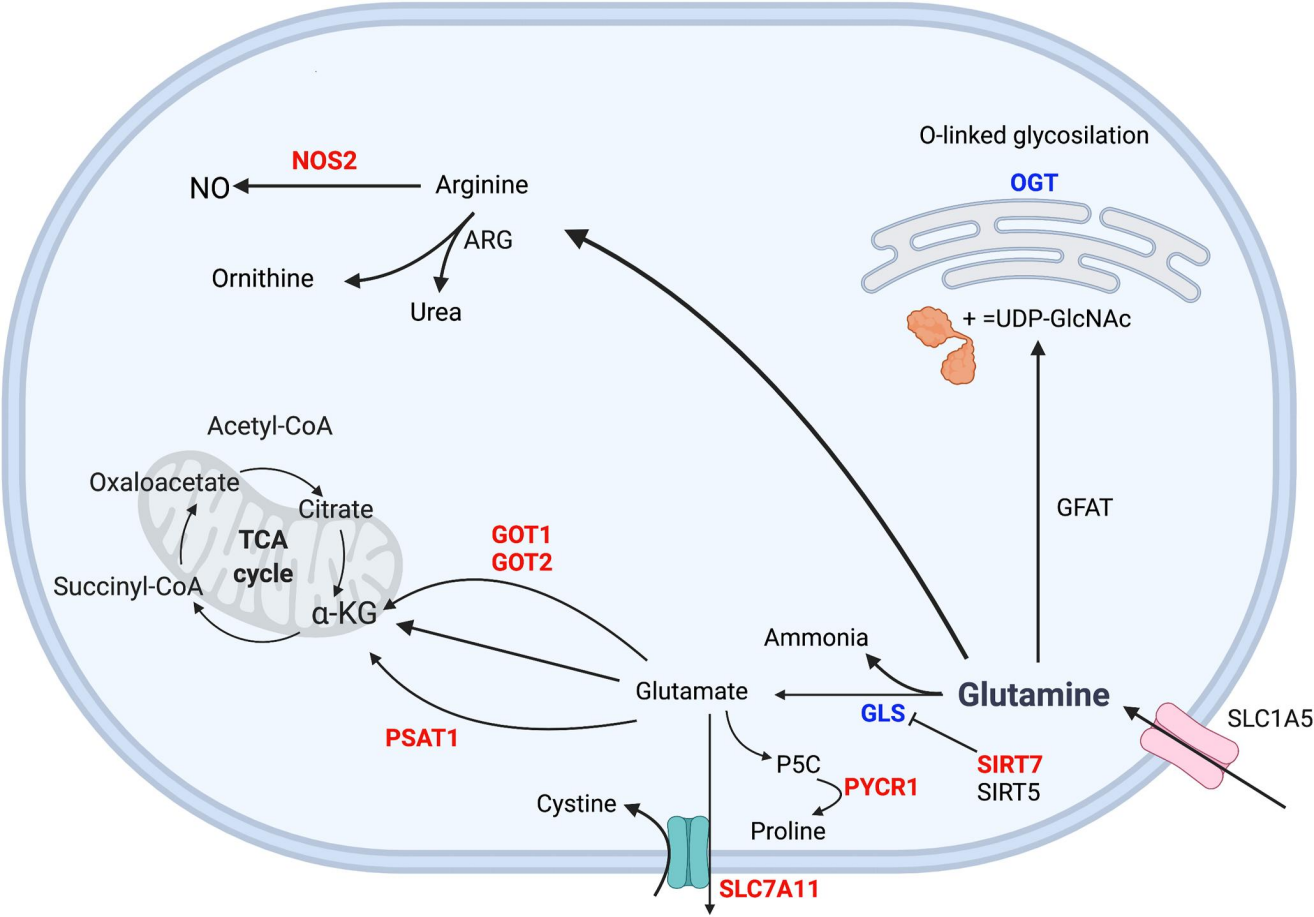
Summary of dysregulated genes involved in glutamine metabolism. In red: genes that are upregulated. In blue: genes that are downregulated. α‐KG, α‐ketoglutarate; Acetyl‐CoA, acetyl coenzyme A; ARG, arginase; GLS, glutaminase; GOT1, glutamic‐oxaloacetic transaminase 1; GOT2, glutamic‐oxaloacetic transaminase 2; NO, nitric oxide; NOS2, nitric oxide synthase 2; OGT, O‐linked N‐acetylglucosamine (GlcNAc) transferase; P5C, pyrroline‐5‐carboxylate; PSAT1, phosphoserine aminotransferase 1; PYCR1, pyrroline‐5‐carboxylate reductase 1; SIRT5, sirtuin 5; SIRT7, sirtuin 7; SLC1A5, solute carrier family 1 member 5; SLC7A11, solute carrier family 7 member 11; TCA cycle, tricarboxylic acid cycle; UDP‐GlcNAc, uridine diphosphate N‐acetylglucosamine

Glutaminolysis is also involved in the generation of proline, an important structural amino acid that has been associated with extracellular matrix remodeling. The aldehyde dehydrogenase 18 family, member A1 (ALDH18A1) gene encodes the enzyme pyrroline‐5‐carboxylate synthase (P5CS), which catalyzes the conversion of glutamate to pyrroline‐5‐carboxylate (P5C). P5C is then converted to proline by PYCR1 using NAD(P)H as a cofactor.^7^ In both datasets analyzed, PYCR1 gene expression was increased in AECs of asthmatics versus HCs. Interestingly, emerging evidence has linked PYCR1 with cell proliferation, apoptosis inhibition, reactive oxygen species production, and inflammation.^8^

As with all studies, there are limitations to our study. Limitations include the fact that gene expression can be discordant with protein level and activity. In addition, it is possible that positive and negative feedback loops might be balancing out some of the observed metabolic abnormalities. Finally, our study does not address causality. Even though we speculate that the dysregulation of glutamine metabolism is an important part of asthma pathogenesis, it is also possible that these abnormalities are the result of airway inflammation. Our group plans to address this critical question by using a novel passive transfection method to knock‐down genes in AECs grown at the air‐liquid interface.^9^ This technique will allow us to study the role of the different genes listed above in the airway epithelial barrier.

In conclusion, there are multiple glutamine metabolism derangements in the airway epithelium of patients with severe and Th2‐high asthma. Further mechanistic studies to dissect the implications of these metabolic abnormalities are warranted.

## AUTHOR CONTRIBUTIONS

**Daniela C. Bravo‐Solarte:** Conceptualization (equal); Data curation (equal); Formal analysis (equal); Investigation (equal). **Kimberly E. Stelzig:** Data curation (equal); Investigation (equal); Methodology (equal); Project administration (equal); **Lyda Cuervo‐Pardo:** Data curation (equal); Formal analysis (equal); Investigation (equal); **Sergejs Berdnikovs:** Formal analysis (equal); Investigation (equal); Methodology (equal); **Sergio Elias Chiarella:** Conceptualization (equal); Data curation (equal); Formal analysis (equal); Funding acquisition (equal); Investigation (equal); Methodology (equal); Project administration (equal).

## FUNDING INFORMATION

National Institute of Allergy and Infectious Diseases, Grant/Award Numbers: K08AI141765; R01AI127783.

## CONFLICT OF INTEREST

The authors have no conflicts of interest to disclose.
